# When Alice Took Sertraline: A Case of Sertraline-Induced Alice in Wonderland Syndrome

**DOI:** 10.7759/cureus.10140

**Published:** 2020-08-30

**Authors:** Maria Vilela, Diana Fernandes, Tatiana Salazar, Cláudia Maio, Augusto Duarte

**Affiliations:** 1 Internal Medicine, Centro Hospitalar do Médio Ave, Vila Nova de Famalicão, PRT; 2 Internal Medicine, Centro Hospitalar Do Médio Ave, Vila Nova de Famalicão, PRT

**Keywords:** alice in wonderland syndrome, body image distortion, sertraline

## Abstract

Alice in Wonderland syndrome (AIWS) is a rare disorder that refers to episodic body image distortions, sometimes associated with altered perception of external space and time. AIWS is mainly associated with viral disease in children as well as migraines and epileptic seizures in adults. Its pathogenesis is still very much unknown and there are not many reported drug-associated cases in medical literature. We describe a case of a 67-year-old woman, with a relevant history of depressive disorder, nontoxic goiter, and dyslipidemia that presented short episodes of altered body perception associated with altered external space perception for a period of one week. Her physical examination was unremarkable and her cerebral computerized axial tomography (cerebral CAT) and blood tests showed no alterations. She had been medicated with sertraline till the previous year and had restarted it in the previous month. She reported similar episodes of body image distortions in the first weeks of initiating sertraline for the first time. The current episode lasted for two weeks and in one year follow-up she reported no recurrence. The patient was diagnosed with AIWS probably induced by sertraline, being the first reported case of the kind.

## Introduction

Alice in Wonderland syndrome (AIWS) is a rare perceptual disorder characterized by body image distortions, altered notion of its position in space and of passing time, often associated with sensations of depersonalization, derealization, and distortions of external objects.

Although first described by the North-American neurologist Caro Lippman in 1952, it was English psychiatrist John Todd who coined the term AIWS in 1955, describing it as a self-experienced paroxysmal body image illusion involving distortions of the size, mass, or shape of the patient’s own body or its position in space, as an almost perfect homage to the protagonist of Alice’s Adventures in Wonderland, written by Lewis Carroll (pseudonym of Reverend Charles Dodgson) [1]. In the book, Alice experiences several changes in her body size and shape (usually after ingesting some food or drink), altered perception of time (as when she is falling through the rabbit hole), and even depersonalization [2].

When first described by Todd, AIWS was present in patients with altered perception of one’s own body image, although some of them could also report altered perception of external space (teleopsia and pelopsia, in which objects look unusually small and distant or large and close, respectively) [1]. However, over the years, the term AIWS was eventually associated with reports of patients that experienced only external space distortions. As these latter reports were much more frequent, there have been a great number of cases erroneously named as AIWS. A recent study by Lanska and Lanska that identified 81 AIWS labeled cases concluded that only 25% met Todd’s original criteria. This concluded that the use of the term AIWS for isolated visual illusions was a problem and should not be continued [3].

The pathogenesis of AIWS is still very much unknown. It is mainly associated with viral diseases in children and with migraines in adults (it is believed that Lewis Carroll suffered from migraines and that the illusions reported by Alice in her adventures are in fact a description of some Carroll’s auras) [3-5]. An Italian review of 2016 shed some light in the neurological pathways involved, with a focus on the temporoparietal-occipital carrefour [6].

The treatment is directed towards the underlined pathology and currently there is no known treatment for the symptoms associated with AIWS. The symptoms themselves are not life-threatening and the episodes usually subside after a few days to weeks.

## Case presentation

We report the case of a 67-year-old woman with a medical history of nontoxic goiter, dyslipidemia and depressive syndrome, medicated with simvastatine 20 mg daily and alprazolam 0.5 mg daily. She had been medicated in the past with sertraline 50 mg daily, but decide to stop the previous year, due to the fact that she felt much better. However, due to increased anhedonia and anxiety in the previous month, she was advised to resume sertraline, which she did in the previous two weeks.

Upon a scheduled medical appointment, she referred that in the previous week she had had episodes of abnormal bodily perceptions. She felt that her hands somehow enlarged or diminished, although she knew that was not possible and when looking they appeared perfectly normal. During these episodes she sometimes felt that her body levitated towards the ceiling. Another time she felt that her whole body grew gigantic and in three occasions she felt instead that she had diminished, as if shrunk by an outside force. Associated to these episodes, the patient reported that some objects (wardrobe, lamp, chair) reduced in size and seemed strangely far away. These episodes lasted only a few minutes and recurred at any given time of day or night. She had insight during the occurrence of all the episodes, referring that she knew what she was describing was just a feeling and that it would be impossible for these alterations to actually occur. She also denied increased anxiety, a sense of detachment, or a feeling of being outside herself during these episodes. Two days previously she was observed by an ophthalmologist and complained about these episodes. Her visual examination showed no alterations from normal standards, and she was advised to consult her attending physician.

A thorough physical examination revealed no alterations and the neurological examination was unremarkable. She was oriented to a revaluation appointment within a month and to pursue investigation a cerebral computerized axial tomography (cerebral CAT) and blood tests (complete hemogram, sodium and potassium concentrations, creatinine and urea, thyroid stimulating hormone, tetraiodotironine and triiodothyronine) were scheduled.

**Figure 1:**
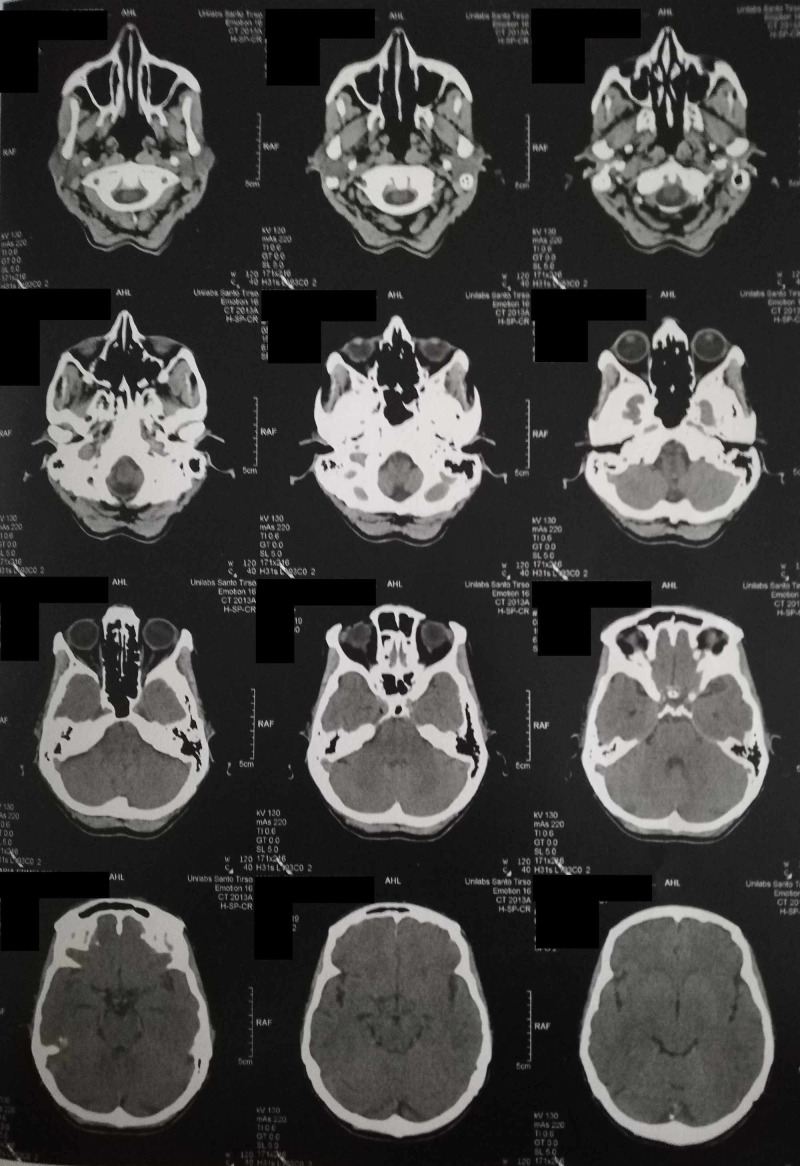
Cerebral CAT. The cerebral CAT is within the normal range for gender and age

**Figure 2:**
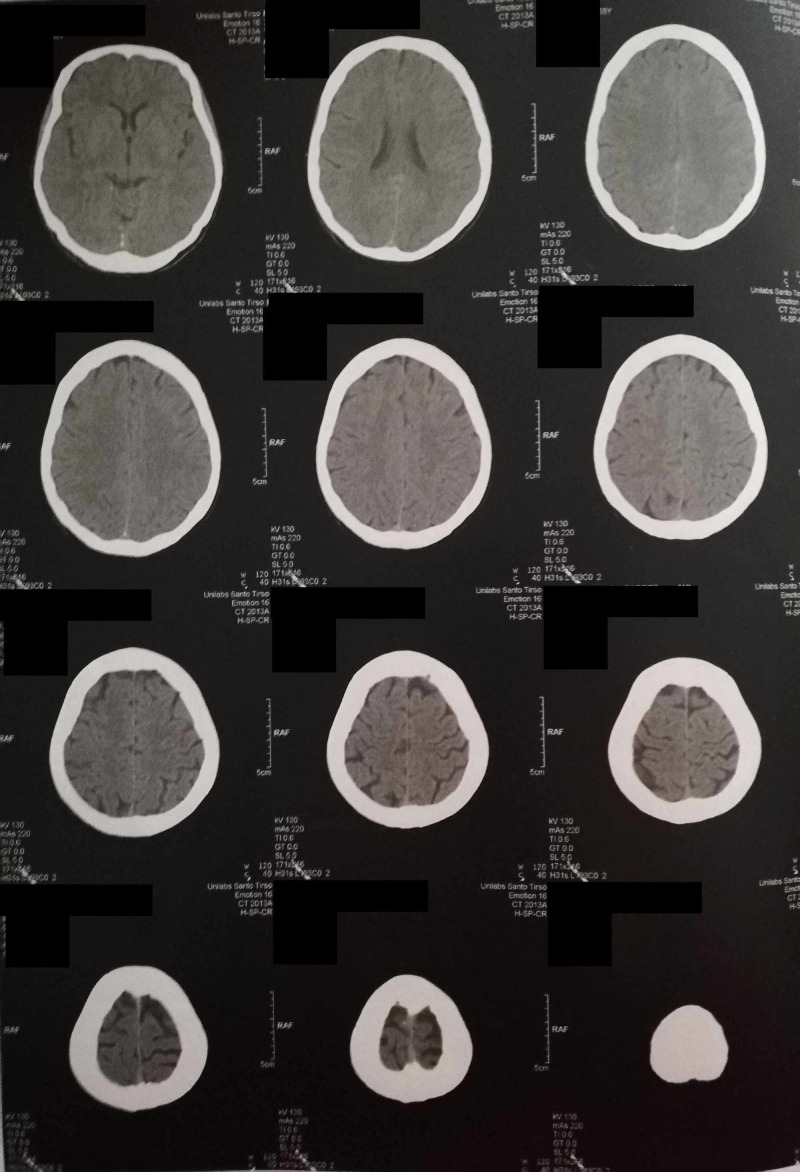
Cerebral CAT. The cerebral CAT is within the normal range for gender and age

One month later she reported that the episodes of abnormal bodily and external perceptions had ceased within a week. She was feeling better from her anhedonia and anxiety and recognized that these improvements were associated with sertraline. However, she remembered that in the first weeks after taking sertraline for the first time, almost ten years ago, she had experienced similar episodes of abnormal bodily sensations, having the sense that she grew or shrunk or even that she levitated. Her blood chemistry, besides a low thyroid stimulating hormone with normal levels of thyroxine and triiodothyronine were unremarkable, the urine screening for drugs was negative, and the cerebral CAT scan showed no alterations (Table 1, Figures 1-2).

**Table 1:** Laboratory workup results.

Analyte	Result
Hemoglobin	15.2 g/dL
Hematocrit	45.80%
Mean corpuscular volume	88.20 fL
Platelets	241 x 10^3^/µL
Leucocytes	4.63 x 10^3^/µL
Neutrophils	66.80%
Lymphocytes	22.00%
Monocytes	6.90%
Eosinophils	3.50%
Basophils	0.60%
Creatinine	0.79 mg/dL
Urea	33 mg/dL
Sodium	142 mEq/L
Potassium	4.6 mEq/L
Calcium (Total)	9.2 mg/dL
Thyroid stimulating hormone	0.005 uUI/mL
Thyroxine (T4)	0.94 ng/dL
Triiodothyronine (T3)	3.77 pg/mL
C reactive protein	< 0.10 mg/dL
Urine screening: amphetamines, methamphetamines, barbiturates, benzodiazepines, cocaine, methadone, opioids, cannabinoids, tricyclic antidepressants	Negative

In a year follow-up, she still reported no new episodes.

## Discussion

The patient was diagnosed with AIWS probably induced by sertraline due to the fact that the reported episodes only occurred when initiating this psychoactive drug. A thorough ophthalmological and neurological examination, as well as a normal blood chemistry and cerebral CAT, excluded alterations that could explain the symptoms and as the patient denied feelings of depersonalization and derealization and as these syndromes could not completely justify the patient’s range of symptoms, they were excluded. A review by Blom thoroughly described a list of pathogenic conditions associated with AIWS, as well as medication and other drugs (ranging from montelukast to cough syrup) and sertraline were not reported. This is probably the first reported case of AIWS that is sertraline induced [7].

## Conclusions

What makes this case unique is that our patient presented with a relatively large number of metamorphopsias and that there are no known reported cases of AIWS due to sertraline. However, AIWS can be frequently misdiagnosed and that may account for the few reported cases of drug-induced AIWS. Not only is this syndrome not frequently mentioned but the difficulty also lies within the patient's reluctance to describe their symptoms out of fear of being labeled with a psychiatric disorder. Nonetheless, it is usually easy to rule out psychosis as those with AIWS have insight. The lack of established diagnostic criteria or large-scale epidemiological studies on AIWS signifies that the exact prevalence of the syndrome is currently unknown.
